# Stationäre Versorgungskosten, kostenverursachende Faktoren und potenzielle Vergütungsprobleme bei Verletzungen und Frakturen im Rahmen epileptischer Anfälle

**DOI:** 10.1007/s00104-020-01257-w

**Published:** 2020-08-05

**Authors:** René D. Verboket, Nils Mühlenfeld, Jasmina Sterz, Philipp Störmann, Ingo Marzi, Yunus Balcik, Felix Rosenow, Adam Strzelczyk, Laurent M. Willems

**Affiliations:** 1 Klinik für Unfall‑, Hand- und Wiederherstellungschirurgie, Goethe-Universität Frankfurt, Theodor-Stern-Kai 7, 60590 Frankfurt am Main, Deutschland; 2 Epilepsiezentrum Frankfurt Rhein-Main und Klinik für Neurologie, Goethe-Universität Frankfurt, Frankfurt am Main, Deutschland; 3 Epilepsiezentrum Hessen und Klinik für Neurologie, Philipps-Universität Marburg, Marburg (Lahn), Deutschland; 4 LOEWE Center for Personalized Translational Epilepsy Research (CePTER), Goethe-Universität Frankfurt, Frankfurt am Main, Deutschland

**Keywords:** Gesundheitsökonomie, Krankheitskosten, DRG, Epilepsie, Lebensqualität, Health economics, Treatment costs, Diagnosis related groups, Epilepsy, Quality of life

## Abstract

**Hintergrund:**

Die Analyse krankheitsspezifischer Kosten gewinnt in einem zunehmend ökonomisch ausgerichteten Gesundheitssystem an Relevanz, wobei vor allem chronische Erkrankungen aufgrund der langen Krankheitsdauer sowie häufiger Hospitalisierung und Arztbesuche von besonderem Interesse sind. Epilepsien stellen eine häufige neurologische Erkrankung dar, welche mit paroxysmal auftretenden epileptischen Anfällen und häufig hiermit assoziierten Verletzungen einhergeht und alle Altersgruppen betrifft.

**Ziel:**

Ziel der Arbeit ist die Aufarbeitung der stationären Behandlungskosten anfallsbedingter Verletzungen sowie die Analyse hinsichtlich relevanter kostenverursachender Faktoren. Mittels alternativer Kalkulation der Versorgungskosten soll zusätzlich der Frage nach potenziellen Vergütungsproblemen im aktuellen DRG-System („diagnosis related groups“) nachgegangen werden.

**Methoden:**

Grundlage dieser monozentrischen, retrospektiven Analyse ist der tatsächliche Erlös der stationären Behandlung von 62 Patienten, die zwischen 01/2010 und 01/2018 im Universitätsklinikum Frankfurt aufgrund von Verletzungen im Rahmen epileptischer Anfälle erfolgte. Die Analyse potenzieller kostenverursachender Faktoren bezog sich auf relevante soziodemographische und klinische Aspekte, die alternative Kalkulation der Versorgungskosten wurde mit gängigen gesundheitsökonomischen Methoden durchgeführt.

**Ergebnisse:**

Der mittlere DRG-Erlös betrug 7408 € (±8993 €, Median 5086 €, Spanne 563–44.519 €), die mittleren kalkulierten Kosten 9423 € (±11.113 €, 5626 €, Spanne 587–49.830 €). Als signifikant kostenverursachender Faktor konnte eine Liegedauer ≥7 Tage (*p* = 0,014) identifiziert werden. Aufgrund des signifikanten Unterschieds (*p* < 0,001) zwischen Erlös und kalkulierten Kosten erfolgte eine Analyse nach Faktoren für potenzielle Vergütungsprobleme, welche für eine Aufenthaltsdauer von ≥7 Tagen (*p* = 0,014) sowie für eine Behandlung auf Intensivstation (*p* = 0,019) signifikant verblieb.

**Schlussfolgerung:**

Die stationären Versorgungskosten von Patienten mit Frakturen aufgrund epileptischer Anfälle sind hoch und daher gesundheitsökonomisch relevant. Generell scheint die auf Fallpauschalen basierende Vergütung nach G‑DRG die tatsächlichen Kosten zu decken, bei Patienten mit einer langen Liegedauer oder einen Aufenthalt auf Intensivstation können jedoch Vergütungsprobleme bestehen.

## Abstract (en)

**Background:**

The systematic analysis of disease-specific costs is becoming increasingly more relevant in an economically oriented healthcare system. Chronic diseases are of particular interest due to the long duration as well as frequent hospitalization and physician visits. Epilepsy is a frequent neurological disorder affecting all age groups with the clinical hallmark of paroxysmal epileptic seizures, which are often associated with injuries.

**Objective:**

The aim of this work was to process the inpatient treatment costs due to seizure-related injuries and fractures. Moreover, relevant cost-causing factors were addressed. Using an alternative calculation of the costs of care, the question of potential reimbursement problems in the current German diagnosis-related groups (G-DRG) system was additionally assessed.

**Methods:**

For this monocentric retrospective analysis the actual proceeds of 62 inpatients who were treated at the University Hospital Frankfurt between January 2010 and January 2018 for injuries and fractures due to epileptic seizures were used. The analysis of potential cost-causing factors was carried out with respect to relevant sociodemographic and clinical aspects. The alternative calculation of the costs of treatment was carried out using established health economic methods.

**Results:**

The average DRG revenue was 7408€ (±8993€, median 5086€, range 563–44,519€), the average calculated costs were 9423€ (±11,113€, 5626€, range 587–49,830€). A length of stay ≥7 days (*p* = 0.014) was identified as a significant cost-driving factor. Due to the significant difference (*p* < 0.001) between revenue and calculated costs, an analysis was made according to factors for potential reimbursement problems, which remained significant for a length of stay of ≥7 days (*p* = 0.014) and for treatment in the intensive care unit (*p* = 0.019).

**Conclusion:**

The inpatient treatment costs for patients with injuries and fractures due to epileptic seizures are high and therefore relevant from a health economic perspective. In general, reimbursement according to the G‑DRG appears to cover the actual costs, but there may be reimbursement problems for patients with a long period of hospitalization or a stay in an intensive care ward.

## Hintergrund

Epilepsie ist eine häufige neurologische Erkrankung, die Patienten jeder Altersklasse betrifft und sich klinisch durch wiederkehrende, meist nichtvorhersagbare epileptische Anfälle unterschiedlicher Semiologie und Ausprägung manifestiert [8, 31]. Im Rahmen epileptischer Anfälle kommt es durch Stürze, Bewusstseinseinschränkungen bzw. unkontrollierbare Muskeltonisierung oder -kloni häufig zu Verletzungen, welche von Hämatomen bis hin zu operativ versorgungspflichtigen Frakturen reichen können [9, 16, 33, 37, 43]. Retrospektiv berichten ca. 14–18 % der Patienten mit Epilepsie über rezente Verletzungen im Rahmen ihrer Anfälle, die Lebenszeitprävalenz anfallsbedingter Verletzungen liegt zwischen 11 und 28 %, wobei eine Häufung von Verletzungen bei Patienten mit therapierefraktärer oder schwer zu behandelnder Epilepsie beschrieben wurde [4, 34, 43]. Insbesondere für Frakturen besteht bei Epilepsiepatienten ein um den Faktor 1,7 bis 6,2 gesteigertes Risiko [39], welches vor allem auf eine reduzierte Knochendichte zurückgeführt wurde [1, 7]. Häufig bedürfen anfallsbedingte Verletzungen einer unmittelbaren medizinischen Versorgung, z. B. Frakturen, Verbrennungen oder Schnitte, weshalb oftmals eine Vorstellung in Notaufnahmen oder neurologischen bzw. unfallchirurgischen Kliniken zwecks weiterer Versorgung erfolgt [37, 43]. Neben den direkten Traumafolgen gehen Verletzungen im Rahmen epileptischer Anfälle mit einer verminderten Lebensqualität der Betroffenen einher, welche eine weitere relevante Belastung für Patienten und deren Angehörige darstellt [21, 37, 42, 43].

Die Analyse krankheitsspezifischer Kosten gewinnt in einem zunehmend ökonomisch ausgerichteten Gesundheitssystem mehr und mehr an Relevanz. Aus gesundheitsökonomischer Sicht setzen sich die verletzungsbedingten Kosten im Rahmen epileptische Anfälle aus drei unterschiedlichen Aspekten zusammen: Neben den direkten Versorgungskosten, z. B. ambulante oder stationäre Versorgung bzw. Heil- oder Hilfsmittel, müssen auch indirekte Kosten, z. B. durch Krankschreibung oder Reduktion der Arbeitskraft, sowie intangible Kosten berücksichtigt werden. Letztere summieren die primär nichtmonetären Folgen von Verletzungen, z. B. eine verminderte Lebensqualität, und versuchen diesen einen monetären Gegenwert zuzuschreiben, was bisher kaum adäquat gelingt [12, 28, 42]. Aus Sicht der Behandler sind insbesondere die direkten, also die im Rahmen der ambulanten oder stationären Versorgung anfallenden Krankheitskosten von Relevanz, da diese durch das G‑DRG(„German diagnosis related groups“)-Vergütungssystem kompensiert werden [24]. Über die letzten Jahre wurde das fallbasierte Vergütungssystem immer wieder kritisiert und auf eine mögliche Diskrepanz zwischen erstatteten und tatsächlich angefallenen Kosten hingewiesen [19, 22, 38].

Ziel dieser Studie ist es, die krankheitsspezifischen Kosten der stationären Versorgung anfallsbedingter Verletzungen aufzuarbeiten, wobei neben der Erfassung des Reinerlöses sowie einer Analyse nach kostenverursachenden Faktoren auch eine alternative Kalkulation der Behandlungskosten nach gängigen gesundheitsökonomischen Methoden erfolgte, um potenzielle Vergütungsprobleme des DRG-Systems zu eruieren.

## Patienten und Methode

### Studiendesign und Datenakquise

Diese verwendeten Daten stammen aus einer retrospektiven Studie mit epileptischen Anfällen assoziierten Verletzungen, die 2019 in der Abteilung für Unfall‑, Hand- und Wiederherstellungschirurgie am Universitätsklinikum Frankfurt durchgeführt wurde. Mittels einer Abfrage im Krankenhausinformationssystem (KIS) nach den ICD-10-Codes G40.0 bis G40.9 der G‑DRG wurden systematisch alle Patienten mit Epilepsie identifiziert, die zwischen Januar 2010 und Januar 2018 stationär in der o. g. Abteilung behandelt wurden. In einem weiteren Schritt wurden alle erfassten Patienten durch zwei unabhängige Untersucher nach akuten Verletzungen im Rahmen epileptischer Anfälle als Vorstellungsursache untersucht. Patienten mit fehlender Assoziation zwischen akuter Verletzung und Anfall wurden ausgeschlossen, ebenso wurde mit unklaren Fällen vorgegangen. Die vorliegende Studie wurde durch die lokale Ethikkommission genehmigt (Frankfurt am Main, Genehmigung 52/18) und folgt den STROBE-Richtlinien für Beobachtungsstudien (Strengthening the Reporting of Observational Studies in Epidemiology) sowie den RECORD-Richtlinien für Observationsstudien (Reporting of Studies Conducted Using Observational Routinely-collected Data; [5, 13]). Ein ähnliches Studiendesign wurde bereits für die gleiche Fragestellung bei Patienten mit Morbus Parkinson verwendet [17, 38].

### Kostenkalkulation

Der DRG-Erlös jedes Falls wurde erfasst und als tatsächlich vergütete Kosten gewertet. Entsprechend den Empfehlungen zur Kalkulation stationärer Behandlungskosten wurde für die alterative Berechnung auf abteilungs- und klinikspezifische Tagessätze zurückgegriffen [3, 15]. Im Jahr 2011 wurden Pauschalen von 593,40 € für Normalstationen und 1337,72 € für Intensivstationen empfohlen [3], welche Pflege, Medikation und Diagnostik beinhalten. Analog zu anderen gesundheitsökonomischen Analysen wurden die Pauschalen für die Jahre 2012 bis 2018 um die Inflation im medizinischen Sektor korrigiert [11, 32, 38, 42]. Die zusätzlich anfallenden Kosten für die operative Versorgung von Frakturen wurden nach Waeschle et al. berechnet, welche im Jahr 2016 die Kosten pro Operationsminute systematisch und DRG-konform analysiert haben. Der vorliegenden Berechnung liegen die über alle Fachabteilungen gemittelten Kosten von 16,63 € pro Minute zugrunde [41], welche ebenfalls um die Inflationsraten im medizinischen Bereich angepasst wurden. Aufgrund der Berechnung krankheitsspezifischer Versorgungskosten nach pauschalisiertem Ansatz wurden, verglichen mit einem Micro-costing-Ansatz, keine räumlichen, apparativen oder personellen Anschaffungs- bzw. Vorhaltekosten berücksichtigt.

### Datenverarbeitung und Statistik

Die Aufarbeitung und statistische Auswertung der Daten erfolgte unter Verwendung von IBM SPSS Statistics 25 (SPSS Inc., Armonk, NY, USA). Die statistische Aufarbeitung der DRG-Erlöse erfolgte aufgrund einer rechtsschiefen Verteilung der anfallenden Kosten unter Verwendung der Bootstrap-Methode [35] unter Angabe von Mittelwert, Standardabweichung, Minimum, Maximum, Median sowie des 95 %-Konfidenzintervalls. Die univariate Analyse von mit höheren Kosten assoziierten Faktoren erfolgte unter einheitlicher Verwendung des Eta-Koeffizienten (η) bei Verwendung je eines metrisch- (Kosten) und eines nominalskalierten Parameters. Aus der Berechnung resultiert ein Koeffizient zwischen 0 (keine Korrelation) und 1 (sehr starke Korrelation). Hierbei zeigt ein η < 0,01 keine Korrelation an, ein η zwischen 0,01 und 0,04 eine geringe, ein η zwischen 0,04 und 0,16 eine mittelstarke und ein η von >0,16 eine starke Korrelation [26]. Zudem erfolgt die Angabe von Somers’ d zur weiteren Beschreibung der Korrelation sowie zur Analyse einer signifikanten Korrelation [18, 26, 30]. Die univariate Analyse hinsichtlich potenzieller Vergütungsprobleme erfolgte in gleicher Weise basierend auf der Differenz zwischen kalkulierten Kosten und tatsächlichem Erlös. Alle Ergebnisse wurden nach der Benjamini-Hochberg-Korrektur für multiples Testen korrigiert [2].

## Ergebnisse

### Studienpopulation, stationärer Aufenthalt, Verletzungen und Versorgung

Von insgesamt 264 Patienten mit Epilepsie als Nebendiagnose in der initialen Erhebung konnten bei 62 Patienten (23,48 %) eine Verletzung im Rahmen eines epileptischen Anfalls als sichere Ursache identifiziert werden. Alle identifizierten Patienten gingen in die finale Auswertung ein. Das Durchschnittsalter der Kohorte betrug 58,1 Jahre (±17,8 Jahre, Median 57 Jahre, Spannweite 21–86 Jahre), 59,7 % waren weiblich (*n* = 37) und 40,3 % männlich (*n* = 25; Tab. 1). Betrachtet man das Verletzungsmuster so zeigte sich mit 40,3 % (*n* = 25) der Fälle ein Schädel-Hirn-Trauma als häufigster Grund für die stationäre Aufnahme und Überwachung. Nachrangig folgend waren Frakturen der oberen Extremität 32,3 % (*n* = 20) und Weichteilverletzungen 30,6 % (*n* = 19) Gründe für stationäre Aufnahmen (Tab. 2). Die mittlere Aufenthaltsdauer im Krankenhaus betrug bei den ausgewerteten Patienten 11,4 Tage (±12,4 Tage, Median 7 Tage, Spannweite 1–64 Tage). Bei den Patienten mit Frakturen konnte in 54,8 % der Fälle (*n* = 34) konservativ vorgegangen werden, 45,2 % (*n* = 28) mussten operativ versorgt werden. In 29,0 % der Fälle (*n* = 18) musste eine Behandlung auf einer Intensivstation erfolgen. Die genauen Verweildauern für Patienten mit und ohne Frakturen auf Normal- und Intensivstationen sind in Tab. 2 dargestellt.

**Table 1:** 

*Geschlecht*	*% (n)*
Weiblich	59,7 (37)
Männlich	40,3 (25)
*Alter *	*(Jahre)*
Mittelwert ± SA	58,1 ± 17,8
Median	57,0
Spannweite	21–86

*SA* Standardabweichung

**Table 2:** 

**Verletzungsmuster**^a^	**% (*n*)**			
Weichteilverletzungen	30,6 (19)			
Fraktur obere Extremität	32,3 (20)			
Fraktur untere Extremität	8,1 (5)			
Fraktur Körperstamm	24,2 (15)			
Schädel-Hirn-Trauma	40,3 (25)			
Fraktur Schädel	9,7 (6)			
**Krankenhausaufenthalt (Tage)**	**MW**	**±SA**	**MED**	**SW**
*Alle Patienten*				
Gesamtdauer	11,4	12,4	7,0	1,0–64,0
Normalstation	10,8	12,2	7,0	1,0–64,0
Intensivstation	1,7	4,2	0,0	0,0–24,0
*Patienten mit Fraktur*				
Gesamtdauer	12,4	13,1	7,0	2,0–64,0
Normalstation	12,2	13,1	7,0	2,0–64,0
Intensivstation	1,4	1,4	0,0	0,0–18,0
*Patienten ohne Fraktur*				
Gesamtdauer	9,2	10,7	2,0	1,0–31,0
Normalstation	7,6	9,6	2,0	1,0–31,0
Intensivstation	2,3	5,7	0,0	0,0–24,0
**Versorgung**	**% (*n*)**			
Aufenthalt Intensivstation	29,0 (18)			
Frakturversorgung operativ	45,2 (28)			
Frakturversorgung konservativ	54,8 (34)			

*MW* Mittelwert, *SA* Standardabweichung, *MED* Median, *SW* Spannweite

^a^Mehrere Verletzungen je Patienten wurden in den einzelnen Gruppen gewertet

### Tatsächlicher Erlös und kalkulierte Kosten

Der tatsächliche mittlere DRG-Erlös der analysierten Fälle betrug 7408 € (±8993 €, Median 5008 €). In der Kalkulation der krankheitsspezifischen Behandlungskosten zeigte sich im Mittel ein Betrag von 9423 € (± 11.113 €, Median 5626 €), wobei der Größte Anteil der angefallenen Kosten der stationäre Aufenthalt aus machte (8916 €, ±11.064 €, Median 4851 €, Tab. 3).

**Table 3:** 

Einzelposten	Kostenberechnung					
	MW (€)	±SA (€)	MED (€)	MIN (€)	MAX (€)	95 %-KI (€)
**Kalkulierte Gesamtkosten Kosten**	9423	11.113	5626	587	49.830	6998–11.749
*Kalkulierte stationäre Versorgung*	8916	11.064	4851	4983	49.830	6517–11.313
Normalstation	6531	7380	4143	587	38.618	4902–8582
Intensivstation	2384	5971	0	0	33.806	1208–3839
*Kalkulierte operative Versorgung*	507	635	0	0	1856	349–656
**DRG Erlös**	7408	8993	5008	563	44.519	5353–9957

*MW* Mittelwert, *SA* Standardabweichung, *MED* Median, *MIN* Minimum, *MAX* Maximum, *KI* Konfidenzintervall

### Univariate Analyse kostenverursachender Faktoren

In der Analyse kostenverursachender Faktoren zeigte sich nach Korrektur für multiples Testen eine starke (η > 0,16) signifikante Korrelation erhöhter direkter Kosten mit einer Aufenthaltsdauer von ≥7 Tage (*p* = 0,014). Eine nicht signifikante, aber starke (η > 0,16) Korrelation erhöhter direkter Kosten fand sich für männliches Geschlecht, ein Alter ≥65 Jahre sowie für eine intensivmedizinische Behandlung, eine nicht signifikante geringe oder mittelstarke (η > 0,04) Korrelation für eine operative Versorgung, eine Fraktur generell sowie der oberen Extremität oder des Körperstamms oder Schädels, eine antikonvulsive Mehrfachtherapie, das Vorliegen einer aktiven Epilepsie sowie eine generalisiert tonisch-klonische Anfallssemiologie. Keine Korrelation (η ≤ 0,04) in Bezug auf die direkten Behandlungskosten fand sich für das Vorliegen eine Schädel-Hirn-Traumas oder das Vorhandensein einer Fraktur der unteren Extremität (s. Tab. 4).

**Table 4:** 

Faktor	Kohorte		Kostenverursachende Faktoren					Potenzielle Faktoren für Vergütungsprobleme				
		% (*n*)	MW (€)	±SA (€)	η^a^	d^a^	*p*-Wert	MW (€)	± SA (€)	η^a^	d^a^	*p*-Wert
*Demographie*												
Geschlecht	w	35,5 (22)	6,818	7,924	0,175	0,122	0,091	3,391	5,066	0,074	0,093	0,434
	m	64,5 (40)	10,856	12,400				4,254	5,979			
Alter	<65	58,1 (36)	7,339	8,549	0,222	0,154	0,115	2,884	566	0,224	0,139	0,220
	>65	41,9 (26)	12,307	13,588				5,420	6,586			
*Versorgung*												
Operative Versorgung	Ja	45,2 (28)	9,995	9,657	0,047	−0,159	0,117	3,424	3,940	0,085	−0,121	0,280
	Nein	54,8 (34)	8,951	12,917				4,379	6,766			
Intensivstation	Ja	29,0 (18)	19,130	14,091	0,563	−0,169	0,117	7,826	7,638	0,787	0,244	*0,019*
	Nein	71,0 (44)	5,452	6,435				2,361	3,642			
Aufenthaltsdauer	<7d	45,2 (28)	2,519	2,150	0,479	0,427	*0,014*	930	909	0,488	0,377	*0,014*
	≥7d	54,8 (34)	15,108	12,295				6,425	6,650			
*Verletzung*												
Fraktur	Ja	69,4 (43)	10,098	10,416	0,092	−0,169	0,117	4,148	5,906	0,054	−0,099	0,405
	Nein	30,6 (19)	7,893	12,749				3,494	5,127			
Fraktur obere Extremität	Ja	32,3 (20)	7,058	6,221	0,151	−0,004	0,974	3,035	3,665	0,114	−0,069	0,465
	Nein	66,1 (42)	10,639	12,876				4,404	6,448			
Fraktur untere Extremität	Ja	8,1 (5)	10,920	10,925	0,040	−0,200	0,807	6,280	8,137	0,123	−0,303	0,640
	Nein	91,9 (57)	9,291	11,226				3,743	5,427			
Fraktur Körperstamm	Ja	24,2 (15)	11,745	10,511	0,119	−0,102	0,280	3,294	4,512	0,066	0,023	0,852
	Nein	75,8 (47)	8,682	11,319				4,156	5,989			
Fraktur Schädel	Ja	9,7 (6)	14,108	17,949	0,139	−0,043	0,465	6,889	10,755	0,172	−0,021	0,807
	Nein	90,3 (56)	9,821	10,260				3,633	4,876			
Schädel-Hirn-Trauma	Ja	40,3 (25)	9,706	14,321	0,021	0,120	0,285	4,646	7,241	0,013	0,084	0,465
	Nein	59,7 (37)	9,321	8,532				3,476	4,298			
*Anfall und Epilepsie*												
Anzahl AED	1	82,3 (51)	8,428	12,286	0,042	0,063	0,465	3,263	4,840	0,057	0,006	0,947
	≥1	17,7 (11)	9,637	10,976				4,096	5,836			
Anfallsfrequenz	≥1/y	66,1 (41)	9,056	4,103	0,046	−0,010	0,974	4,103	5,650	0,046	−0,10	0,940
	<1/y	33,9 (21)	10,139	13,268				3,644	5,760			
Generalisierter Anfall	Ja	80,6 (50)	9,751	11,640	0,061	−0,027	0,807	4,180	5,952	0,085	−0,051	0,569
	Nein	19,4 (12)	8,055	8,944				2,980	4,196			

*MW* Mittelwert, *SA* Standardabweichung

^a^Korrelationsanalyse mittels η und Somers’ d nach Schäfer und Schöttker-Königer [26] und Somers [30], signifikante *p*-Werte nach multiplem Testen (*p* < 0,05) in Kursivschrift

### Univariate Analyse potenzieller Faktoren für Vergütungsprobleme

Die univariate Analyse nach potenziellen Faktoren für Vergütungsprobleme zeigte eine signifikante, starke (η > 0,16) Korrelation der Diskrepanzen zwischen kalkulierten Kosten und tatsächlichem Erlös in Bezug auf eine Aufenthaltsdauer von >7 Tagen (*p* = 0,014) sowie für einen Aufenthalt auf Intensivstation (*p* = 0,019). Eine starke (η > 0,16), nicht signifikante Korrelation fand sich für ein Alter ≥65 Jahre sowie für eine Fraktur des Schädels. Eine nicht signifikante geringe oder mittelstarke (η > 0,04) Korrelation zeigte sich für männliches Geschlecht, eine operative Versorgung, das Vorhandensein einer Fraktur generell sowie für eine Fraktur der oberen und unteren Extremität und des Körperstamms sowie für eine antikonvulsive Mehrfachtherapie, eine Anfallsfrequenz von ≥1 in den letzten 12 Monaten und das Vorhandensein einer generalisiert tonisch-klonischen Anfallssemiologie (s. Tab. 4).

## Diskussion

Die Analyse krankheitsspezifischer Kosten gewinnt in einem mehr und mehr ökonomisch ausgerichteten Gesundheitssystem zunehmend an Relevanz. Diese Tendenz einer Ökonomisierung der Medizin wurde über die letzten Jahre kontrovers diskutiert und kritisiert [14, 27, 29, 40]. Unabhängig davon, ob man diese Tendenzen als positiv oder negativ betrachtet, resultiert aus der zunehmenden Ökonomisierung der medizinischen Versorgung insbesondere für die erstversorgenden Instanzen, z. B. Notaufnahmen, Ambulanzen oder Kliniken, ein intrinsisches und existenzielles Interesse, die tatsächlich angefallenen Kosten durch die fallbezogene Vergütung nach G‑DRG-System zu decken. Immer wieder wurde beschrieben, dass in Bezug auf spezielle Krankheitsbilder die tatsächlichen Kosten durch den DRG-Erlös nicht zwangsläufig zu decken sind [22, 38], was die Kalkulation der tatsächlich anfallenden Kosten zu einer zentralen gesundheitsökonomischen Aufgabe macht [38, 42].

Gesundheitsökonomisch besonders relevant ist hierbei die Erfassung der Kosten häufiger chronischer Erkrankungen, da diese im Hinblick auf die Gesamtausgaben des Gesundheitssystems einen beträchtlichen Teil ausmachen. Die krankheitsspezifischen Kosten für neurologische Erkrankungen beliefen sich im Jahr 2019 laut Statistischem Bundesamt auf 17,2 Mrd. €, wovon wiederum 1,6 Mrd. € (9,3 %) auf Patienten mit Epilepsie bzw. epileptischen Anfällen zurückzuführen sind [6]. Hierbei teilen sich die krankheitsspezifischen Kosten, welche zuletzt mit 14.500 € pro Jahr für Patienten mit aktiver Epilepsie und 2500 € für anfallsfreie Patienten pro Jahr kalkuliert wurden, in etwa zu gleichen Teilen auf direkte und indirekte Kosten auf, wobei ein Trend über die letzten Jahre hinsichtlich eines zunehmenden Anteils indirekter Kosten zu beobachten ist [20, 42, 44].

Ein häufiger notfälliger Vorstellungsgrund von Patienten mit Epilepsie oder einem epileptischen Anfall in unfallchirurgischen oder orthopädischen Kliniken sind in der Regel anfallsassoziierte Unfälle und Verletzungen („epilepsy related injuries and accidents“ [ERIA]), für welche eine jährliche Prävalenz von 14–18 % und eine Lebenszeitprävalenz zwischen 11 und 28 % beschrieben wurde [4, 43]. Aufgrund einer hohen Dunkelziffer fälschlich nicht kausal auf epileptische Anfälle zurückgeführte Verletzungen ist von einer deutlich höheren Gesamtprävalenz auszugehen [10], was die ökonomische Relevanz anfallsbedingter Verletzungen unterstreichet.

Der tatsächliche mittlere DRG-Erlös der untersuchten Kohorte belief sich auf 7408 €, was bei einer mittleren Verweildauer von 12 Tagen 617 € Erlös pro Tag bedeutet. Hierbei konnte eine signifikante Korrelation der Verweildauer mit dem Erlös nachgewiesen werden. Die alternative Kalkulation der angefallenen krankheitsspezifischen Kosten mittels Tagessätzen und Verweildauer auf Intensiv- und Normalstation sowie minutengenauer Vergütung der Operationsdauer, wenn zutreffend, ergab einen Betrag von 9423 €, entsprechend 785 € pro Tag, entsprechend einer Differenz von täglich 168 €, entsprechend einem signifikanten Unterschied zum tatsächlichen Erlös. Die Analyse hinsichtlich potenzieller Faktoren, die diese Abweichung bedingen und somit ein Vergütungsproblem darstellen könnten, zeigte eine signifikante Diskrepanz insbesondere bei einer stationären Verweildauer von ≥7 Tagen sowie einem Aufenthalt auf Intensivstation. Bezüglich einer längeren Aufenthaltsdauer als ein potenzieller kostenverursachender Faktor passt das vorliegende Ergebnis mit einer rezenten Untersuchung der stationären Versorgungskosten bei Patienten mit Frakturen im Rahmen von Stürzen bei idiopathischem Parkinson-Syndrom sowie weiteren gesundheitsökonomischen Arbeiten überein [25, 36, 38, 45]. Dies mag prinzipiell auf einen Zusammenhang von Vergütungsproblemen beim Überschreiten der Obergrenze der mittleren Verweildauer hinweisen. Das Prinzip der mittleren Verweildauer mit einer Obergrenze wurde bereits kurz nach Einführung der DRG-Pauschalen auf dem Gebiet der Unfallchirurgie und Orthopädie deutlich kritisiert und Nachbesserungen bei der Vergütung gefordert [23]. Obwohl diese zwischenzeitlich erfolgt sind, erscheint insbesondere bei langer Liegedauer bei Patienten mit Verletzungen durch epileptische Anfälle jedoch weiterhin ein Vergütungsproblem zu bestehen. Auch die Diskrepanz bezüglich der Vergütung von Patienten mit intensivmedizinischer Behandlung wurde bereits generell sowie bei anderen Krankheitsbildern beschrieben [22] und verweist auf eine mögliche Unterrepräsentation dieser Fälle in der aktuellen pauschalisierten Vergütung nach G‑DRG. Im Hinblick auf beide Aspekte sind weitere versorgungsmedizinische Analysen notwendig, um ggf. eine weitere Anpassung der Vergütung begründen zu können.

Trotz Beachtung der STROBE- und RECORD-Richtlinien für Beobachtungsstudien bzw. Observationsstudien unterliegt diese Studie aufgrund ihres Designs potenziellen Limitationen [5, 13]. Neben der begrenzten Anzahl an analysierten Patienten, welche jedoch in Anbetracht des orientierenden Charakters der Studie ausreichend erscheint, schränkt auch das retrospektive, monozentrische Design der Studie die Interpretation der Daten ein. Die Anpassung der Ausgangskosten für die alternative Kalkulation der Versorgungskosten durch Anpassung nach Inflation im medizinischen Sektor folgt den aktuell gebräuchlichen Methoden, verzerrt die Kosten jedoch mit zunehmendem Abstand zur primären Erhebung. Aufgrund des für die Berechnungen verwendeten auf Tagespauschalen und Operationsminuten basierenden Ansatzes konnten, verglichen mit einem prospektiven Micro-costing-Ansatz, keine gesonderten räumlichen, apparativen, materiellen oder personellen Ausgaben berücksichtigt werden. Ebenso wurden keine generellen Anschaffungs- bzw. Vorhaltekosten berücksichtigt, da diese in Bezug auf krankheitsspezifische Versorgungskosten nicht von Interesse sind, sich in der DRG-Vergütung aber ebenfalls widerspiegeln sollten. Aufgrund der einheitlichen Vergütung stationärer Leistungen in Deutschland nach G‑DRG sowie der standardisierten SOP- und leitlinienorientierten Behandlung von Frakturen erscheint ein Vergleich der Ergebnisse mit anderen Kliniken auf nationaler Ebene jedoch vertretbar.

Zusammenfassend sind die stationären, direkten Versorgungskosten von Patienten mit Verletzungen im Rahmen epileptischer Anfälle hoch und aufgrund ihrer Häufigkeit gesundheitsökonomisch relevant. Die auf Fallpauschalen basierende Vergütung der Kosten scheint überwiegend kostendeckend zu sein, analog zu bereits veröffentlichten Untersuchungen scheinen insbesondere bei Patienten mit langer Liegedauer oder intensivmedizinischer Versorgung Vergütungsprobleme zu bestehen. Weitere gezielte gesundheitsökonomische Analysen, z. B. mittels exakter Erfassung der einzelnen Kosten („micro-costing“) werden benötigt, um diesen Hinweisen weiter nachzugehen und eine verbesserte Kostendeckung medizinischer Maßnahmen durch das aktuelle, auf Fallpauschalen basierte Vergütungssystem nach G‑DRG zu erreichen.
